# Modeling the combined impact of water activity and temperature on *Salmonella* Montevideo thermal inactivation on dried chili peppers

**DOI:** 10.1111/1750-3841.70201

**Published:** 2025-04-17

**Authors:** Natoavina T. Faliarizao, Jemel Fanfan, Narindra Randriamiarintsoa, Muhammad Siddiq, Teresa M. Bergholz, Kirk D. Dolan

**Affiliations:** ^1^ Department of Food Science and Human Nutrition Michigan State University East Lansing Michigan USA; ^2^ Department of Medicine Columbia University New York New York USA; ^3^ Department of Biosystems and Agricultural Engineering Michigan State University East Lansing Michigan USA

**Keywords:** capsicum, mathematical modeling, *Salmonella*, thermal resistance, water activity

## Abstract

*Salmonella* contamination in low‐moisture foods remains a continuing food safety and public health problem. The World Health Organization ranked unprocessed red chili peppers as the spice with the highest risk of *Salmonella* contamination in a risk assessment‐based report in 2022. Therefore, appropriate mitigation strategies are required to control *Salmonella* contamination in dried chilies. The objectives of this research were to investigate thermal inactivation of *Salmonella* on dried chili peppers at *a_w_* 0.33–0.97 and estimate thermal resistance parameters under isothermal and dynamic conditions. The dried chili peppers were inoculated with *S*. Montevideo and conditioned in a humidity‐controlled chamber to achieve the *a_w_* of 0.33, 0.50, and 0.97. The samples were placed into 1‐mm thick aluminum test cells and heated at 55, 60, 65, and 70°C to estimate the thermal inactivation parameters using the log‐linear/modified‐Bigelow model. The results showed that the *a_w_* played a key role in accurately describing *Salmonella* lethality on dried chili pepper. At 65°C, a 3‐log reduction of *S*. Montevideo required 100 min at *a_w_* = 0.33, compared to only 20 min at *a_w_* = 0.50. The modified Bigelow model under dynamic conditions described inactivation significantly better (root mean squared error [RMSE] = 0.646 and corrected Akaike information criterion [AIC_c_] = −120.97) than the modified Bigelow model under isothermal conditions (RMSE = 0.707 and AIC_c _= −76.71). This study provides the food industry with valuable data to optimize thermal processing conditions and improve safety protocols for chili‐based products, thereby reducing the risk of *Salmonella* contamination.

## 1 INTRODUCTION

Spices and herbs are low‐moisture ingredients used in food preparation for their flavor‐enhancing properties. Spices, often used in small quantities, can harbor a variety of pathogens and spoilage microorganisms, which can lead to foodborne illnesses. Although the low‐moisture content of dried spices can inhibit the growth of most non‐spore‐forming microorganisms, *Salmonella* can survive in such conditions for months (Erdoğdu & Ekiz, 2011; Xie, 2022). *Salmonella* spp. contamination was responsible for 95% of food recalls associated with spices in the United States between 1969 and 2003, underscoring the severity of this problem (Zhang, Hu, et al., 2017). Recent data showed that, between 2012 and 2020, herbs and spices represented 4.9% of the total low‐moisture food recalls (Acuff et al., 2023). Furthermore, Eissa et al. (2024) reported that, between 2000 and 2022, *Salmonella* contamination was the first cause of recalls in spices and herbs. Pathogen contamination is often due to the vulnerability of the product to environmental exposure (insects, wild animals, or foreign materials) during traditional sun drying and improper food handling during production (Bakobie et al., 2017; Fudholi et al., 2014; Kara et al., 2015). *Salmonella* in spices has been documented in several countries, indicating an ongoing global problem with implications for food safety (Bedada et al., 2018; Gieraltowski et al., 2013; Zweifel & Stephan, 2012).

The World Health Organization (2022) ranked red chili pepper processed under suboptimal conditions as the spice and herb with the highest risk of *Salmonella* contamination. Multiple methods of spice decontamination exist, including steam treatment, gases (propylene oxide and ethylene oxide raise consumer concerns), radio‐frequency processing, and milder alternatives, such as chlorine dioxide, ozone, and hydrogen peroxide (Wason et al., 2021). Spices, such as dried chili, are often added directly to uncooked or ready‐to‐eat foods (Xie, 2022), for example, fermented uncured salami, pizza, and pasta garnish, thus creating potential food safety risks in case of *Salmonella* contamination. Dried chili peppers (*Capsicum annuum* L.) are increasingly used as low‐moisture ingredients in multiple ready‐to‐eat foods and are a popular spice due to their pungent taste and bright red color. In the United States, 272 people from 44 states suffered from salmonellosis between 2009 and 2010 as a result of eating salami containing black and red pepper that was contaminated with *Salmonella* (CDC, 2010; Gieraltowski et al., 2013). Therefore, developing appropriate handling, storage, and pathogen control strategies for chili peppers is crucial to prevent the risk of *Salmonella* contamination and ensure food safety.

Foodborne pathogens, including *Salmonella*, can survive in various low‐water activity products (Casulli, Igo, et al., 2021; Grasso‐Kelley et al., 2021; Randriamiarintsoa et al., 2024; Smith, 2014; Verma et al., 2021; Xie et al., 2021). The concept of water activity (*a_w_*) was introduced as a quantitative approach to understanding microbial response in foods (Labuza & Altunakar, 2020; López‐Malo & Alzamora, 2015). Smith (2014) found that the rate of desiccation or hydration had no impact on *Salmonella* thermal resistance; rather, the *a_w_* at the time of thermal treatment played a key role. *Salmonella* thermal resistance increases with decreasing moisture, emphasizing the impact of *a_w_* on pathogen survival (Hildebrandt et al., 2024; Podolak et al., 2010). In addition, low *a_w_* in spices poses a challenge in eliminating pathogens without compromising quality (Beuchat et al., 2013). Therefore, research is needed to understand the effects of *a_w_* on *Salmonella* thermal resistance, which has led to its inclusion in existing predictive inactivation models.

Multiple modeling approaches have been developed to describe the thermal inactivation of *Salmonella* in low‐moisture foods, including spices. Models incorporating the influence of *a_w_* on thermal inactivation on spices are limited, and Smith et al. (2016) reported that the log‐linear/modified Bigelow model showed superior parameter estimation ability when combining the effect of *a_w_* and temperature. However, most studies on thermal inactivation of *Salmonella* use isothermal conditions (including the ones incorporating the effect of *a_w_*) that do not reflect commercial food processing, where the product temperature and *a_w_* are dynamically changing, as occurs during food drying. Therefore, the main objectives of this study were to investigate the *Salmonella* thermal inactivation on dried chili peppers over a wide range of *a_w_* (0.33, 0.50, and 0.97) and to estimate *Salmonella* thermal resistance parameters under isothermal and dynamic conditions. The higher *a_w_* level was included to offer additional understanding of *Salmonella* survival during chili drying processes.

## 2 MATERIALS AND METHODS

### 2.1 Chili pepper samples

The dried, crushed red chili peppers were purchased from Regal Spices. To verify whether the chili flakes were uncontaminated by *Salmonella*, a 1‐g sample was incubated in 500 mL Luria‐Bertani (LB) broth at 37°C for 24 h. After incubation, the sample was transferred to modified TSA (soybean tryptic agar with 0.05% ammonium citrate and 0.03% sodium thiosulfate) and incubated at 37°C for 48 h. The absence of *Salmonella* in chili samples was confirmed by the use of non‐selective differential media, that is, modified TSA (limit of detection: 1 log_10_ CFU/g). The formation of a black precipitate in the center of the colonies, which is characteristic of *Salmonella* colonies, enables *Salmonella* to be distinguished from other bacteria (Smith et al., 2016).

### 2.2 Experimental design

**FIGURE 1:**
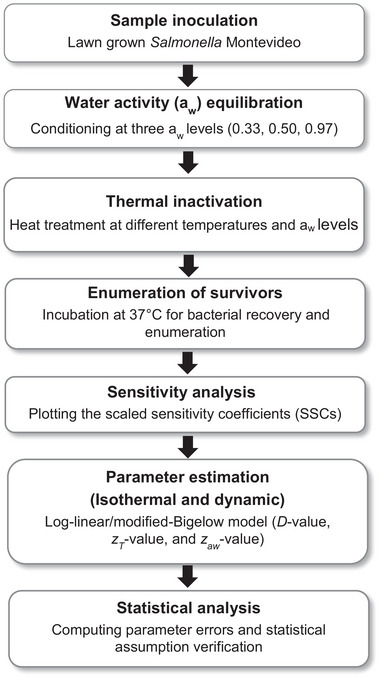
Study design for the mathematical modeling for thermal inactivation of *Salmonella* Montevideo on chili peppers under isothermal and dynamic conditions.

A two‐factor randomized experimental design was used, and all experiments were performed using three biological replicates. The *Salmonella enterica* Montevideo strain (Food Safety Laboratory [FSL] R8‐3881, clinical isolate) was chosen because it was found to be resistant in red pepper powders at different humidity levels (Lee et al., 2022) and was associated with a nationwide outbreak of black and red pepper contamination that affected 272 individuals in 44 states in 2009 and 2010 (Gieraltowski et al., 2013). This strain was acquired from the FSL at Cornell University. This research examined the thermal resistance of *S*. Montevideo across a range of *a_w_* values. Inoculated samples were conditioned to three *a_w_* values (0.33, 0.50, and 0.97) and then subjected to heat treatments at different temperatures (Figure 1). The *Salmonella* population was counted to assess the survival under each treatment (*n* = 3). The log‐linear/modified‐Bigelow model (Smith et al., 2016) was used for the analysis of the thermal inactivation curves. Statistical analysis then evaluated the parameter accuracy and the model assumptions.

### 2.3 Lawn preparation and dried chili pepper inoculation

The *S*. Montevideo strain was maintained at −80°C using tryptic soy broth medium supplemented with 20% (v/v) glycerol. Prior to use, culture was transferred twice in LB broth media and maintained at 37°C for 20 h. The broth culture (250 µL) was then placed on plates (100 × 15 mm^2^) composed of TSA to achieve a uniform growth lawn. After a 24‐h incubation at 37°C, the bacterial lawn was collected using a sterile L‐shaped spreader and suspended in 2.2 mL of 0.1% peptone water to create an inoculum.

To achieve a target inoculation of 7.5‐8.0 log_10_ CFU/g for dried chili peppers, 1.0 mL of the starting inoculum was used to inoculate 100 g of dried chili pepper sample for each biological replicate. The inoculum suspension was placed in a sterile bag with the uninoculated dried chili peppers and shaken manually for 4–5 min to mix the contents. After mixing, the pepper flakes were placed in a sterile stainless‐steel tray and mixed with a sterile spatula for 1 min. To check for uniformity after inoculation, four randomly selected inoculated samples of 2.5 g each were enumerated. The inoculum was considered evenly distributed when the standard deviation (SD) of the randomly selected samples was <0.5 log_10_ CFU/g.

### 2.4 Water activity equilibration

Before each heat treatment, the *a_w_* of each inoculated sample was equilibrated to a target level. The inoculated dried red chili peppers, weighing about 100 g, were put into 100 × 15 mm^2^ Petri dishes and placed inside a custom‐made *a_w_* conditioning apparatus to control the *a_w_* (Smith et al., 2016). This system included a custom control operation in addition to an equilibration chamber (69 × 51 × 51 cm^3^). The control system included a moisture monitoring device inside the chamber, solenoid faucets, an air flow system, a hydration column, a desiccation column, and a computer‐based monitoring operation to maintain the relative humidity (RH) level. The samples were conditioned for 2–3 days in the conditioning apparatus at variable RH levels. The *a_w_* at 25°C was checked every 12 h using a water activity meter (model 4TE, Aqualab); the target *a_w_* of 0.33, 0.50, and 0.97 was achieved.

### 2.5 Thermal inactivation treatment

The thermal inactivation kinetic data collection is based on a thermal‐death time (TDT) test that involved two factors (temperature and *a_w_*) and a 3 × 3 factorial design experimental setup. The three fixed *a_w_* values in this design were 0.33, 0.50, and 0.97 at 25°C, along with constant water bath temperatures of 55, 60, 65, and 70°C. To ensure repeatability, every experiment was conducted with three biological replicates. The heat treatments were performed using 1‐mm thick aluminum test cells as described by Chung et al. (2008). Approximately 0.5 g samples of inoculated and *a_w_* equilibrated dried chili flakes were placed inside these cells. A circulating water bath (Vesta Precision) with a temperature setting of 55, 60, 65, or 70°C was also used to submerge the cells. Uninoculated samples were placed in test cells with a T‐type thermocouple installed in the center of each cell to measure chili sample temperature. Time 0 for the isothermal treatment was designated by measuring the sample's core temperature when it approached the target temperature by 0.5°C, which is known as the come‐up time and can range from 50 to 70 s. Beginning with the samples taken at Time 0, treated chili flakes were extracted at nine equally spaced time points. At each time point, three test cells (two with inoculated chili flakes and one with uninoculated flakes and a thermocouple) were removed from the water bath and quickly placed in an ice‐water bath for 40 s to stop the thermal inactivation process.

### 2.6 Enumeration of viable bacteria

To count the number of *S*. Montevideo survivors after TDT test, the heat‐treated chili peppers (0.5 g) were taken from the test cells and placed in sterile 15 mL centrifuge tubes. Samples were then diluted with 4.5 mL of 0.1% peptone water containing 0.5% (w/v) potassium sulfite (K_2_SO_3_) (Sigma‐Aldrich) to a dilution ratio of 1:10. K_2_SO_3_ was used as a neutralizing agent for the antimicrobial compounds in chili peppers that may affect the pathogen enumeration (Lins, 2018; Xie, 2022). The diluted samples were then homogenized using a vortex at 3000 rpm for 60 s. Serial dilutions were then conducted with 0.1% peptone water in a ratio of 1–10. The dilutions were periodically plated onto modified TSA. After the plates were incubated at 37°C for 24 h, the number of *S*. Montevideo colonies, which are represented by black spots on the plates, was counted and log_10_ transformed for each biological replicate. Log reductions were calculated by subtracting the initial log population from the log survival numbers.

### 2.7 Sensitivity analysis using simulation

To evaluate the estimability of parameters, a scaled sensitivity coefficient (SSC) analysis was performed using a simulated experiment as described by Dolan and Mishra (2013). The sensitivity coefficient (*X*′) indicates how much the response variable changes in response to parameter changes (Beck & Arnold, 1977).

For microbial inactivation modeling, the SSC is measured in log*N* units. Briefly, if the maximum absolute value of the SSC is less than 5% of the maximum log*N* value, the parameter is considered insignificant and remains constant, so no estimation is required. For a parameter to be estimated, its SSC must be large and uncorrelated with others. If a parameter does not meet either condition, it is assigned a predefined value based on literature or experiments. If two parameters are highly correlated, estimation is done separately rather than simultaneously (Dolan & Mishra, 2013).

For each *a_w_* level, the SSC analysis was based on a simulated experiment. Because each treatment was conducted at a constant temperature, the SSC for *z_T_* was not large, as *z_T_* can only be accurately estimated when there is temperature variation. Therefore, a simulated dynamic experiment was conducted in MATLAB to represent SSCs. The temperature was set to increase linearly:

(1) $$T_{simulated}=T_{\min}+t\cdot \frac{\left(T_{\max}-T_{\min}\right)}{t_{\max}}$$

where *T*_min_ is the lowest temperature of the chili pepper sample (24.5°C); *T*_max_ denotes the highest water bath temperature (70°C); *t*_max_ is the duration of the longest experiment, that is, the lowest temperature water bath (5.8, 50, and 200 min for *a_w_* = 0.97, *a_w_* = 0.50, and *a_w_* = 0.33, respectively). The secant forward‐difference method was used in MATLAB (Chapra, 2018).

### 2.8 Mathematical modeling

#### 2.8.1 Bigelow model for isothermal conditions

The primary model selected for the isothermal conditions was the logarithmic‐linear model by Schaffner and Labuza (1997):

(2) $$\log N=\log N_{0}-\frac{t}{D(T)}$$

where log*N* is the population at time *t*; log*N*_0_ represents the initial microbial population; and *D*(*T*) denotes the *D*‐value, which is the time required to reduce the microbial population by one log unit (90% reduction) at a specific temperature *T*.

In isothermal conditions, the temperature at the center of the test cell (*T*(*t*)) was adjusted to match the target water bath temperature for each treatment. The initial population (log*N*_0_) represents the population at the come‐up time when *T*(*t*) was equal to the target temperature.

For the secondary model, the *D*‐value variation with temperature *T* was characterized using the Bigelow model:

(3) $$D\left(T\right)=D_{ref}\cdot 10^{\left(\frac{T_{ref}-T\left(t\right)}{z_{T}}\right)}$$

where *z_T_* represents the *z_T_*‐value, which is the temperature increase needed to reduce the *D*‐value by a factor of 10 (1‐log reduction). T(*t*) is the product temperature (°C); *D*_ref_ denotes the reference *D*‐value at a certain *T*_ref_; and *T*_ref_ represents the optimized reference temperature, at which the correlation between D_ref_ and *z_T_* is the lowest.

By inserting Equation (3) in Equation (2), the Bigelow model for isothermal inactivation is obtained:

(4) $$\log N=\log N_{0}-\left(\frac{t}{D_{ref}}\right)\cdot 10^{\left(\frac{T\left(t\right)-T_{ref}}{z_{T}}\right)}$$

where *D*_ref_ represents the reference *D*‐value at the optimized reference temperature condition, *T*_ref_ = 70°C. Under the isothermal conditions, the product temperature at the center of the test cell labeled *T*(*t*) was set to the target water bath temperature for each treatment.

To model the combined effect of temperature and *a_w_* on the heat resistance of *S*. Montevideo on chili peppers, the modified Bigelow‐type linear model (Gaillard et al., 1998) was used:

(5) $$D\left(T,a_{w}\right)=D_{ref}\cdot 10^{\left(\frac{T_{ref}-T}{z_{T}}\right)}\cdot 10^{\left(\frac{{a_{w}}_{,ref}-a_{w}}{z_{a_{w}}}\right)}$$

where *T*_ref_ represents the optimized reference temperature; *a_w_* is the product water activity; *a_w_*_,ref_ denotes the optimized reference *a_w_*; and *D*_ref_ denotes the *D*‐value at the *T*_ref_. *T*(*t*) is the treatment temperature; *z_T_* represents the *z_T_*‐value; and *z_aw_* represents the *z_aw_*‐value, which is the *a_w_* change needed to reduce the *D*‐value by 10‐fold.

The following equation represents the final modified Bigelow model for linear temperature and *a_w_* conditions:

(6) $$\log N=\log N_{0}-\left(\frac{t}{D_{ref}}\right)\cdot 10^{\left(\frac{a_{w}\left(t\right)-a_{w{,}_{ref}}}{za_{w}}+\frac{T\left(t\right)-T_{ref}}{z_{T}}\right)}$$

The log*N*_0_, *D*_ref_‐value*, z_T_*‐value, and *z_aw_*‐value were globally estimated using the *nlinfit* function in MATLAB. Although the log*N*_0_ is controlled within a certain range, the log*N*_0_ can contain experimental errors as the log*N*, which could be due to natural (inoculum) and experimental (operator) variabilities. Van Boekel (1996) noted that the initial concentration should be treated as normal datum points because of these experimental variabilities. All the temperature‐time history data and the estimated *z_T_*‐values corresponding to each *a_w_* were used in the model. To reduce the error in the *D*_ref_ parameter, the reference temperature, *T*_ref_, was optimized by minimizing the absolute value of the correlation coefficient among *D*_ref_‐_,_ *z_T_*‐, and *z_aw_*‐values.

For comparison, all *D*‐values corresponded to the *D*‐values at the reference conditions, *T*_ref_ = 70°C and *a_w,_*_ref_ = 0.987. The final thermal resistance parameters (*D*_ref_, *z_aw_*, and *z_T_*) were estimated globally to describe *S*. Montevideo inactivation during isothermal conditions.

#### 2.8.2 Bigelow model for dynamic conditions

**FIGURE 2:**
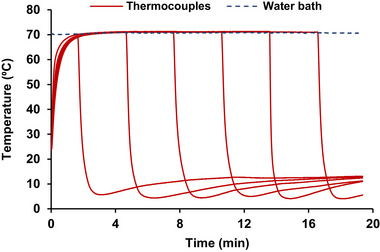
Example of time‐temperature profile of thermal treatment of *Salmonella* Montevideo on chili peppers under dynamic conditions (water bath temp = 70°C at *a_w_* = 0.50).

A comparison of model fitting of isothermal conditions and dynamic conditions was performed to understand the effect of dynamic temperature conditions on the model accuracy. In contrast to isothermal conditions, *T*(*t*) changed dynamically under dynamic conditions and was measured using a thermocouple instead of being fixed at a constant value (Figure 2). The initial population (log*N*_0_) represents the *S*. Montevideo population at the beginning of the experiment, which is before reaching the come‐up time.

For dynamic temperature conditions, given a linear relationship between the *S*. Montevideo population (log*N*) over time, the model can be expressed in differential form as follows:

(7) $$\frac{d(\log N)}{dt}=-\frac{1}{D(T)}$$

By inserting Equation (3) in Equation (7) and integrating both sides, we obtain the final model for dynamic conditions:

(8) $$\log N=\log N_{0}-\left(\frac{1}{D_{ref}}\right)\int_{0}^{t}10^{\left(\frac{T\left(t\right)-T_{ref}}{z_{T}}\right)}dt$$

where the term $\int_{0}^{t}10^{\left(\frac{T(t)-Tr}{z_{T}}\right)}$ is the time‐temperature history for a particular dynamic experiment. The dynamic temperature at the center of the test cell is denoted by *T(t)*. At each *a_w_*, the ordinary least squares non‐linear regression was used to simultaneously estimate *D*_ref_, *z_T_*, and log*N*_0_ using all data simultaneously with full time‐temperature histories in MATLAB. As an alternative to the traditional two‐step approach of model fitting, the one‐step approach provides more accurate estimated parameters with reduced uncertainty (Dolan & Mishra, 2013).

The following equation represents the final model with dynamic temperature and *a_w_* conditions:

(9) $$\begin{array}{c} \log N=\log N_{0}-\left(\frac{1}{D_{ref}}\right)\int_{0}^{t}10^{\left(\frac{a_{w}\left(t\right)-a_{w{,}_{ref}}}{z_{a_{w}}}+\frac{T\left(t\right)-T_{ref}}{z_{T}}\right)}dt \end{array}$$

The time‐temperature‐*a_w_* history is represented by

(10) $$\int_{0}^{t}10^{\left(\frac{a_{w}\left(t\right)-a_{w{,}_{ref}}}{z_{a_{w}}}+\frac{T\left(t\right)-T_{ref}}{z_{T}}\right)}$$

where *T(t)* represents the dynamic temperature of the chili flakes at the center of the test cells and *a_w_(t)* represents the *a_w_* of the sample. *T*_ref_ and *a_w_*_,ref_ are the reference temperature and reference *a_w_* optimized to minimize the error in the *D*_ref_ parameter. The data from the dynamic temperature conditions for all the *a_w_* were utilized for the one‐step approach using *nlinfit* in MATLAB. Surface plots were developed based on the modified Bigelow model under isothermal and dynamic conditions to estimate the *D*‐value in Equation (5) using MATLAB.

### 2.9 Comparison of models and statistical analysis

Statistical analysis is typically performed at the end of mathematical modeling and represents a critical step. First, the normality of the residuals can be confirmed using a residual histogram that overlaps with the standard error of the residuals, and the probability density function of a normal distribution with mean zero. The second regression hypothesis that needs to be evaluated is the independence of the residuals. To achieve this, the residuals can be plotted against the estimated values (Georgalis et al., 2023). If the model's assumptions are correct, the plot should not reveal any clear trends. The third regression hypothesis that needs to be evaluated is the homoscedasticity, that is, whether the variance of the residuals is constant or not. Another recommended tool for evaluating this hypothesis is a plot of residuals versus estimated values (Georgalis et al., 2023).

The root mean squared error (RMSE) is the criterion used to check the goodness of fit of the models. To avoid positive and negative deviations canceling each other out, the RMSE is a measure of absolute error that squares these deviations. The parameter value that best fits the predicted values to the observed data is indicated by the lowest RMSE:

(11) $$RMSE=\sqrt{\frac{\sum {\left(y_{obs}-y_{pred}\right)}^{2}}{n-p}}$$

where *n* is for the number of observations, *p* is the number of parameters to estimate, *y*_pred_ is the predicted values, and *y*_obs_ is the observed values.

The corrected Akaike information criterion (AIC_c_) between estimated and experimental values was used to assess the fit quality of the two models to the experimental data. A lower AIC_c_ indicates a better fit:

(12) $$\mathrm{AI}C_{c}=n\cdot \ln\left(\frac{\mathrm{SSR}}{n}\right)+2\cdot K+\frac{2\cdot K\left(K+1\right)}{n-K-1}$$

where *n* is the number of observed data, SSR denotes the sum of squared residuals, and *K* denotes the number of estimated parameters in the model plus 1 (*p* + 1). AIC_c_ is a tool to support whether adding parameters to a model is associated with a decrease in the sum of squares of residuals.

All statistical analysis for bacterial enumeration was performed using SAS 9.4. ANCOVA (*α* = 0.05) was used to determine the survival of *S*. Montevideo using time as a covariate.

## 3 RESULTS AND DISCUSSION

### 3.1 Dried chili pepper inoculation

*S*. Montevideo survived in dried chili pepper, with an initial population of 7.86 ± 0.48 log_10_ CFU/g. The SD of inoculation was less than 0.5 log_10_ CFU/g, indicating that the inoculum level in all samples was consistent. Xie (2022) used a similar inoculation technique with a higher initial inoculum concentration (∼11 log_10_ CFU/mL), at a ratio of 1 mL per 100 g of spices, particularly dried chili peppers, cinnamon, and black peppers, and yielded an initial *Salmonella* population of 9 log_10_ CFU/g with an SD of <0.5 log_10_ CFU/g. After the inoculation, the samples were conditioned for 72, 48, or 48 h to reach the target water activity of 0.33, 0.50, or 0.97, respectively, prior to the thermal treatment. *S*. Montevideo populations decreased by an average of 0.25 ± 0.1 log_10_ CFU/g after *a_w_* conditioning, which was not significant (*p* > 0.05). Lin et al. (2023) reported a similar population reduction of *Salmonella* on low‐moisture foods during *a_w_* conditioning.

Introducing *Salmonella* into low‐moisture foods creates a stressful environment that potentially leads to higher bacterial reduction when treatments are applied immediately after inoculation (Finn et al., 2013; Wason et al., 2021). However, allowing the bacteria to adapt by equilibrating the inoculated food for a few days is essential for accurate challenge studies. Verma et al. (2021) found that *Salmonella* and *Enterococcus faecium* populations remained stable in dried basil leaves from Day 5 to 15, and similar stabilization was observed with black peppercorns before ethylene oxide treatment (Wei et al., 2021). Thermal treatments applied immediately after inoculation often achieve higher bacterial reductions due to the lack of time to adapt to the desiccated environment (*a_w_* < 0.70), which increases bacterial stress and enhances reduction (Blessington et al., 2013; Jeong & Kang, 2014). Equilibration mimics commercial conditions, as adapted bacteria exhibit higher thermal resistance (Jin et al., 2018), improving the relevance of challenge study outcomes. Initial microbial concentration (log*N*_0_) presents potential experimental variabilities and can be considered a model parameter for the thermal inactivation model as demonstrated by several researchers (Cattani et al., 2016; Dolan, 2003; Dolan et al., 2013; Van Boekel, 1996).

### 3.2 Modeling thermal resistance of *S*. Montevideo

#### 3.2.1 Isothermal conditions

*S*. Montevideo showed more thermal resistance on dried chili pepper with lower *a_w_*, requiring higher temperatures and longer treatment times (*p* < 0.05). At 65°C, a 3‐log reduction required 100 min at *a_w_* = 0.33, compared to only 20 min at *a_w_* = 0.50. Similarly, an over nine fold decrease of D_70°C_ of *S*. Enteritidis PT30 in cinnamon was observed between *a_w_* = 0.2 and *a_w_* = 0.5 (Xie et al., 2021). The thermal resistance of *S*. Montevideo increased with decreasing *a_w_* (*p* < 0.05), which poses a significant challenge for the elimination of pathogens in low‐moisture foods such as chili peppers and spices. Beuchat et al. (2013) noted that low *a_w_* in foods and ingredients with low moisture content, including spices, poses a challenge in eliminating pathogens without compromising quality. Furthermore, Podolak et al. (2010) noted that the heat resistance of *Salmonella* increases with decreasing humidity, highlighting the influence of *a_w_* on pathogen survival during processing.

**FIGURE 3:**
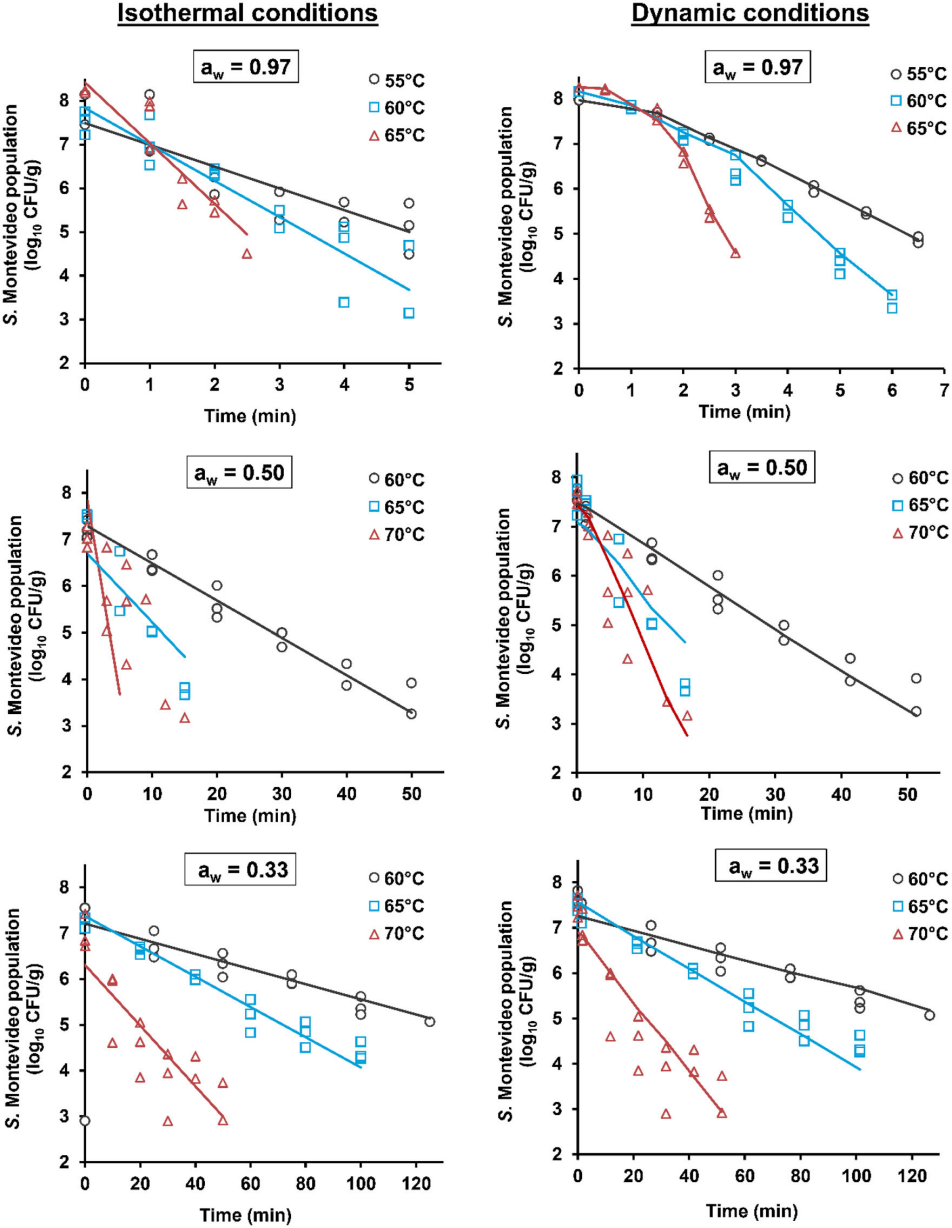
Survival curves for *Salmonella* Montevideo on red chili peppers (n = 3) using the linear (isothermal) and non‐linear (dynamic) Bigelow model at different aw levels and temperatures. The solid line for each survival curve denotes the estimated values.

The Bigelow model for isothermal conditions (Equation 4) was fitted to each of the constant *a_w_* data sets (Table 1). Model fitting results are presented in Figure 3 by plotting lines with experimental measurement markers. For isothermal conditions, at *a_w_* = 0.33, *D*‐values decreased sharply with increasing temperature, from 60.49 min at 60°C to 15.11 min at 70°C, with moderate error percentages (7.39%–14.33%). The corresponding *z_T_*‐value was 16.60°C, indicating higher thermal sensitivity, and the model demonstrated strong performance with an RMSE of 0.465 log_10_ CFU/g and AIC_c_ = −66.27. Similar *D*‐values for *Salmonella* at *a_w_* = 0.33 were reported for chili powder and cinnamon powder with *D*_70°C_‐values of 15.4 and 20.8 min, respectively (Xie, 2022). Verma et al. (2021) noted that the *D*_75°C_‐value for *Salmonella* in dried basil leaves was 9.14 min at *a_w_* 0.40. At *a_w_* = 0.50, *D*‐values were notably lower, ranging from 12.49 min at 60°C to 3.67 min at 70°C, and the *z_T_*‐value increased to 18.42°C with a slightly higher error of 14.31%. The model retained good accuracy with an RMSE of 0.585 log_10_ CFU/g, although the AIC_c_ increased to −27.95, indicating a less optimal fit compared to *a_w_* = 0.33. At *a_w_* = 0.97, *D*‐values were the lowest (2.01 min at 55°C to 0.72 min at 65°C), but the *z_T_*‐value increased to 22.44°C, reflecting reduced thermal sensitivity. This condition exhibited the highest error percentage (21.26%), though the model fit remained acceptable (RMSE = 0.539 log_10_ CFU/g).

**TABLE 1:** Thermal inactivation kinetics of *Salmonella* Montevideo on chili peppers with different equilibrated *a_w_* (0.33, 0.50, and 0.97) at different temperatures under isothermal conditions.

*a_w_*	Temp (°C)	*D*‐value (min)	95% CIs	Error (%)	*z_T_*‐value (°C)	95% CIs	Error (%)	RMSE (log_10_ CFU/g)	AIC_c_
0.33	60	60.49	43.01–77.97	14.33	16.60	11.80–21.40	14.27	0.465	−66.27
	65	30.24	25.72–34.74	7.39					
	70	15.11	12.06–18.16	9.99					
0.50	60	12.49	9.69–15.29	10.96	18.42	13.32–24.33	14.31	0.585	−27.95
	65	6.78	5.63–7.92	8.28					
	70	3.67	2.69–4.65	13.04					
0.97	55	2.01	1.44–2.57	13.78	22.44	12.76–32.12	21.26	0.539	−41.52
	60	1.20	1.02–1.38	7.24					
	65	0.72	0.54–0.90	12.37					

Abbreviations: AIC_c_, corrected Akaike information criterion; CIs, confidence intervals; RMSE, root mean squared error.

#### 3.2.2 Dynamic conditions

Under dynamic conditions, *D*‐values denoted similar trends as under isothermal conditions, with improved model accuracy (Table 2). At *a_w_* = 0.33, the *D*‐values were significantly higher (88.11 min at 60°C) than under isothermal conditions (*p* < 0.05), and the *z_T_*‐value was lower (12.96°C), indicating increased thermal sensitivity. The model demonstrated exceptional precision with an error of 7.57%, an RMSE of 0.400 log_10_ CFU/g, and an AIC_c_ of −97.84. At *a_w_* = 0.50, *D*‐values and *z_T_*‐values (18.02°C) were consistent with isothermal results but showed improved precision (error < 10%), whereas model fit remained comparable (RMSE = 0.554 log_10_ CFU/g, AIC_c_ = −41.80). For *a_w_* = 0.97, the *D*‐values were slightly higher, and the *z_T_*‐value was significantly lower (15.47°C) compared to isothermal conditions, with improved precision (error around 10%) and better model fit (RMSE = 0.496 log_10_ CFU/g, AIC_c_ = −60.03). Isothermal models, which assume static moisture levels, do not consider dynamic changes in moisture content during dynamic processes such as drying, which significantly impact heat resistance. This can lead to an overestimated lethality of the process, which can lead to errors in the validation of preventive control measures (Dolan & Mishra, 2013). Therefore, using parameters estimated from isothermal experiments to predict *Salmonella* inactivation in commercial thermal processes with gradual warming can result in significant underestimates of survival.

**TABLE 2:** Thermal inactivation kinetics of *Salmonella* Montevideo on chili peppers with different equilibrated *a_w_* (0.33, 0.50, and 0.97) at different temperatures under dynamic conditions.

*a_w_*	Temp (°C)	*D*‐value (min)	95% CIs	Error (%)	*z_T_*‐value (°C)	95% CIs	Error (%)	RMSE (log_10_ CFU/g)	AIC_c_
0.33	60	88.11	67.86–108.36	11.45	12.96	10.99–14.92	7.57	0.400	−97.84
	65	36.24	31.94–40.54	5.91					
	70	14.91	13.29–16.52	5.39					
0.50	60	13.57	10.80–16.34	10.07	18.02	14.01–22.02	10.98	0.554	−41.80
	65	7.16	6.24–8.08	6.37					
	70	3.78	3.10–4.46	8.85					
0.97	55	2.22	1.76–2.67	10.21	15.47	11.93–19.00	11.32	0.496	−60.03
	60	1.05	0.94–1.16	5.30					
	65	0.50	0.40–0.59	9.69					

Abbreviations: AIC_c_, corrected Akaike information criterion; CIs, confidence intervals; RMSE, root mean squared error.

The dynamic models have been successfully applied in various commercial food processes. These dynamic processes included dry roasting of peanuts (Casulli, Igo, et al., 2021), dry heating (Valdramidis et al., 2006), roasting of cocoa (Yan et al., 2021), beef jerky drying (Yoon et al., 2006), garlic drying (Zhang, Qi, et al., 2017), hot air heating of pistachios (Casulli, Dolan, et al., 2021), and vacuum steam pasteurization of wheat grain (Lin et al., 2023). The models used for these dynamic processes included log‐linear, Weibull, dynamic sigmoidal, or logistic models for primary models and Bigelow, modified Bigelow, response surface, or Arrhenius for secondary effects.

#### 3.2.3 Combined effect of temperature and water activity (a_w_)

The use of dynamic conditions improved the mathematical modeling accuracy of *S*. Montevideo thermal inactivation on dried chili peppers. Both modified secondary Bigelow models for isothermal conditions (Equation 6) and dynamic conditions (Equation 9) were fitted to describe *Salmonella* thermal inactivation under different *a_w_* levels (Table 3). The model fit for the dynamic temperature data (RMSE = 0.646 log_10_ CFU/mL and AIC_c_ = −120.97) was better than the isothermal data (RMSE = 0.707 log_10_ CFU/mL, and AIC_c_ = −76.71). The *D*‐ and *z*‐values (*z_T_* and *z_aw_*) were more accurately represented by the dynamic parameter results, with parameter relative errors ranging from 1.45% to 12.25% (Table 3). Similarly, Hassani et al. (2005) noted that the model predictions for heat inactivation of *Listeria monocytogenes* under dynamic treatments fit the measured data well regardless of the magnitude of the thermotolerance increase. Furthermore, the comparison of the dynamic model's estimated values and the observations accurately described the linear inactivation pattern (Trevisani et al., 2017), thereby strengthening the dynamic models’ effectiveness in dynamic settings. The reason being, the *S*. Montevideo inactivation follows a log‐linear pattern; however, the non‐isothermal profile affects the inactivation rate, resulting in a curved response, particularly at high temperature and *a_w_*.

**TABLE 3:** Parameter estimates for isothermal and dynamic conditions of *Salmonella* Montevideo in red chili peppers for in‐container thermal treatment at (*T*_ref_ = 70°C, *a_w,_*_ref_ = 0.33).

	Isothermal				Dynamic			
Parameter	Estimate	Error (%)	RMSE (log_10_ CFU/g)	AIC_c_	Estimate	Error (%)	RMSE	AIC_c_
*D*_70°C_‐value (min)	17.61	10.63	0.707	−76.71	16.11	12.25	0.646	−120.97
*z_T_*‐value (°C)	24.03	15.73			16.25	8.14		
*z_aw_*‐value	0.44	2.91			0.38	2.14		
log*N*_0_ at 70°C (log_10_ CFU/g)	6.35	2.98			7.04	1.45		

Abbreviations: AIC_c_, corrected Akaike information criterion; RMSE, root mean squared error.

The *z_aw_*‐values (*a_w_* change required for a 10‐fold change in *D*‐value) of *S*. Montevideo in chili peppers ranged between 0.38 and 0.44 at 70°C, which is a higher value than what had been previously reported in the literature. For instance, the *z_aw_*‐values for *S*. Enteritidis PT 30 in ground cinnamon were 0.33 at 70°C and 0.31 at 75°C (Xie et al., 2021). Water adsorption isotherms and *a_w_* changes during heat treatment vary depending on the food matrix, potentially influencing *Salmonella* inactivation (Gautam et al., 2020). Several studies have investigated the influence of *a_w_* and temperature on the thermal resistance of *Salmonella* in low‐moisture foods. Research on milk chocolate, ground cinnamon, soy protein powder, wheat flour, peanut butter, pet food pellets, and black pepper powder consistently shows that *Salmonella*’s thermal resistance increases as *a_w_* decreases (Gautam et al., 2020; Jin et al., 2020; Smith, 2014; Sun et al., 2023; Syamaladevi et al., 2016; Xie et al., 2021). For milk chocolate, the *z_aw_*‐values were 0.42 at 70°C, 0.36 at 75°C, and 0.46 at 80°C. The relationship between *D*‐values and *a_w_* is generally exponential or semi‐logarithmic (Jin et al., 2020; Sun et al., 2023). Higher temperatures reduce the thermal resistance of *Salmonella*, but the effect is less pronounced at lower *a_w_* levels (Jin et al., 2020; Sun et al., 2023).

**FIGURE 4:**
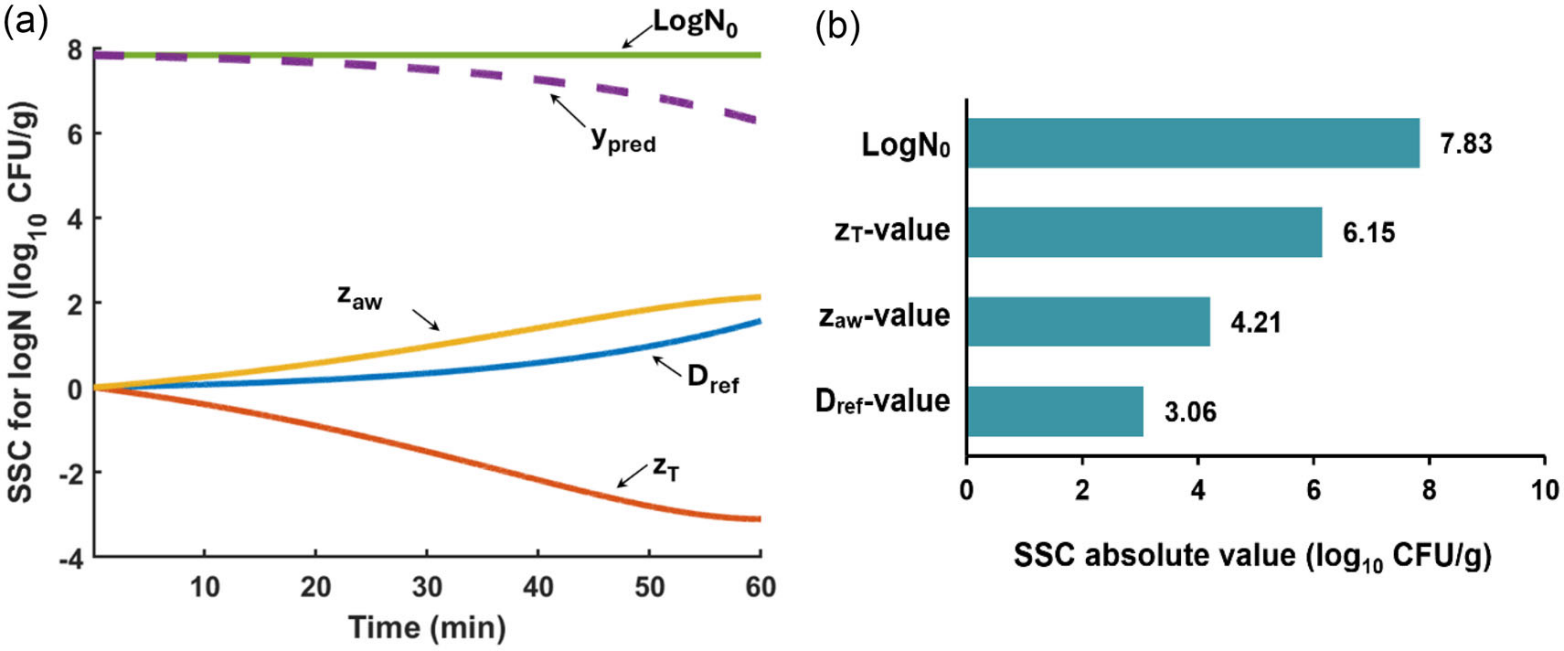
(a) Scaled sensitivity coefficient (SSC) analysis of the thermal inactivation parameters of *Salmonella* Montevideo on chili peppers; (b) tornado plot denoting the influence of parameters.

The SSC plots were essential for identifying which parameters can be estimated most accurately to describe the combined effect of temperature and *a_w_* on *Salmonella* thermal inactivation. For all treatments, the parameter that is estimated with the highest accuracy is log*N*_0_ because its SSC is the largest (maximum value = 7.83) and uncorrelated with the SSC of the other parameters (Figure 4a,b). The SSC for the *z_T_*‐value is the next largest SSC, with a maximum absolute value of 6.15. For the *D*_ref_‐value, the maximum absolute value of SSC is 3.06, which is the lowest of all the parameters. The ratio of SSCs for *D*‐ and *z*‐values was not constant throughout treatment, demonstrating noncorrelation. The model parameters (*D*_ref_, *z_T_*, *z_aw_*, and log*N*_0_) were found to be uncorrelated based on the SSC analysis, which enabled a one‐step parameter estimation approach. Fitting error accumulation is reduced by applying a one‐step regression analysis to all of the isothermal data (Valdramidis et al., 2005). In this study, the optimized reference temperature was 70°C, which was close to the upper end of the temperature range.

**FIGURE 5:**
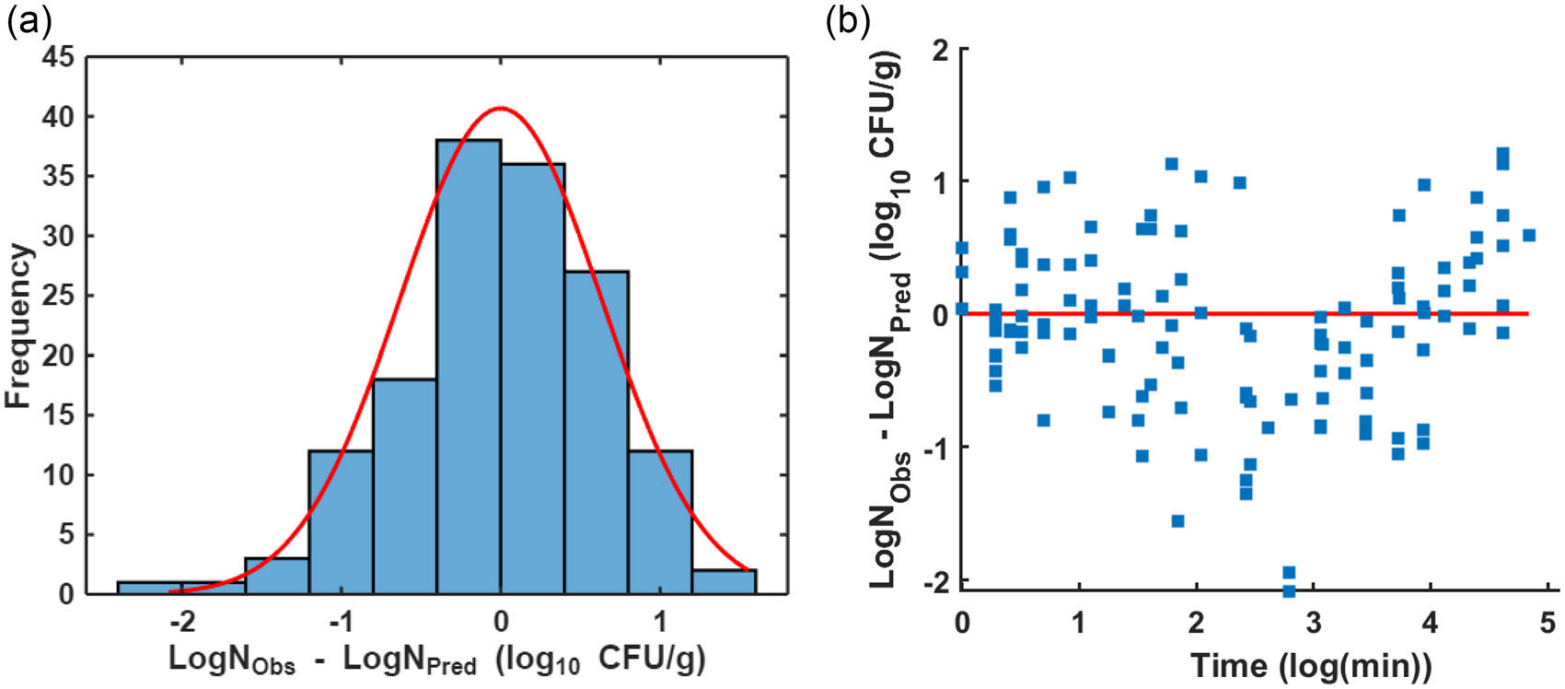
(a) Histogram of the residuals and (b) estimated versus experimental *Salmonella* Montevideo survivors over time for the modified Bigelow model.

**FIGURE 6:**
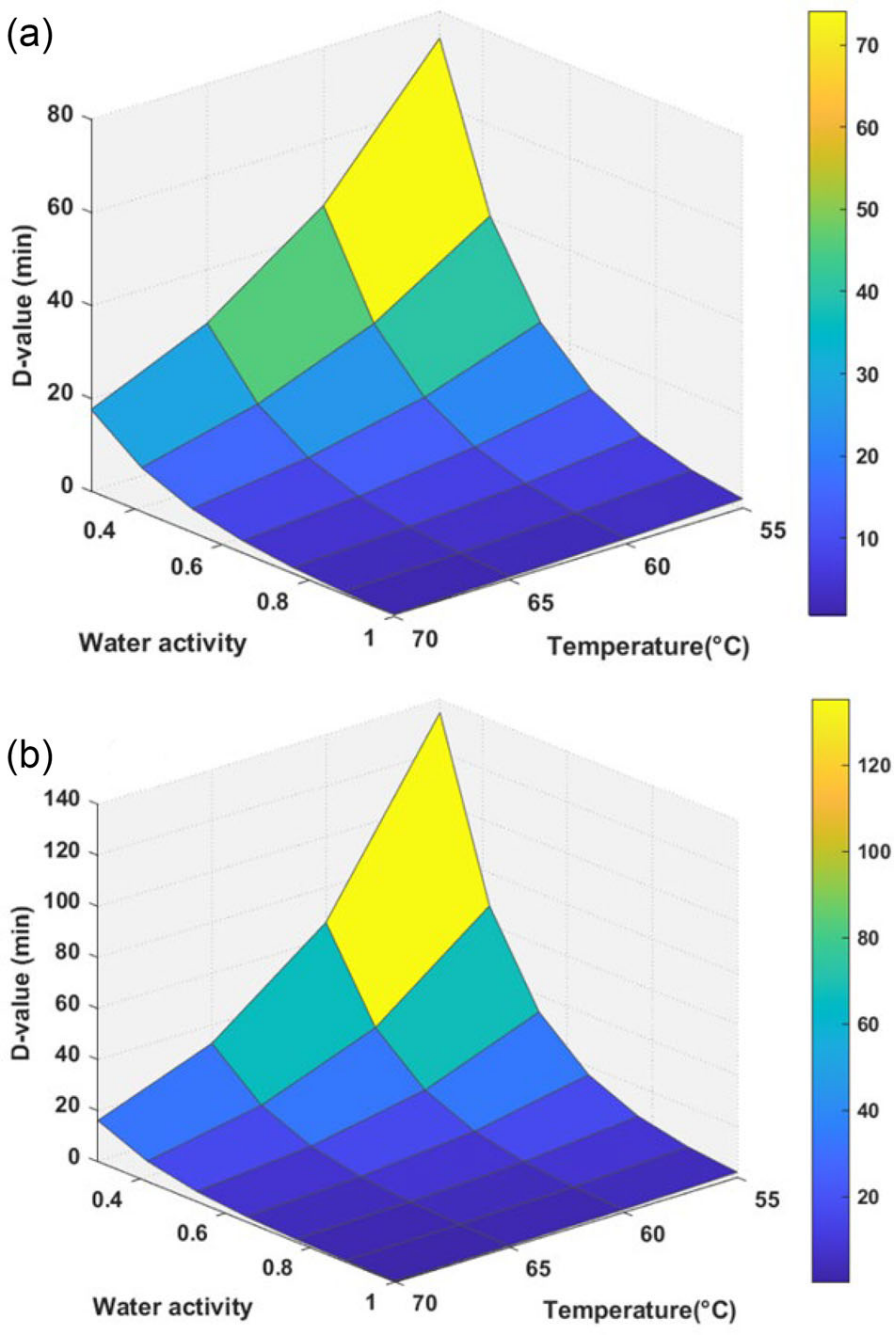
Surface plots showing the *D*‐values as affected by *a_w_* and temperature for *Salmonella* Montevideo using (a) the modified Bigelow model under isothermal conditions and (b) the modified Bigelow model under non‐isothermal conditions.

Residual analysis of the *S*. Montevideo inactivation curves revealed an almost normal distribution, confirming the effectiveness of the model used to estimate pathogen inactivation in dried chili peppers (Figure 5a). Larger residuals were observed during the first half of the treatment, suggesting adaptation challenges initially, but at other heating times the residuals followed an additive pattern with constant variance and a mean close to zero (Figure 5b). This suggested room for improvement in both data collection and the model and emphasized the importance of residual analysis for parameter estimation (Dolan & Mishra, 2013). In addition, the temperature and a_w_ demonstrated a positive effect on *S*. Montevideo inactivation, as shown in the surface plots (Figure 6), which were developed to estimate D‐values.

The findings demonstrate the significance of considering dynamic conditions in modeling to increase parameter estimation accuracy and better replicate real‐world scenarios. Food processing is a multivariable, nonlinear, dynamically complex process where the temperature and the *a_w_* can change and can be controlled to result in increased pathogen lethality. Min et al. (2002) demonstrated that increasing *a_w_* enhances the effectiveness of pulsed electric field treatment in inactivating *Enterobacter cloacae* in chocolate liquor. Lang et al. (2017) developed models for heat inactivation of foodborne pathogens in milk powder, emphasizing *a_w_* as a crucial parameter in thermal decontamination. Carlson et al. (2005) found that decreasing meat *a_w_* from 0.99 to 0.95 reduced the rate of *Salmonella* thermal inactivation by 64% in ground turkey. Smith (2014) investigated the thermal resistance of *Salmonella* in wheat flour, concluding that product *a_w_* at the time of thermal treatment, rather than desiccation or hydration history, determines pathogen resistance. Hildebrandt et al. (2024) demonstrated that single‐level moisture‐based prediction models, which resulted in overestimation of *Salmonella* lethality in the baking of crackers, are inappropriate for describing inactivation in dynamically changing temperature and moisture. These studies collectively underscored the importance of considering *a_w_* in pathogen inactivation models, as it significantly influences the effectiveness of various decontamination methods across different food matrices.

## 4 CONCLUSION

The findings of this study showed that *S*. Montevideo persisted at low water activity dried chili peppers. *Salmonella* showed higher heat resistance in low water activity foods, making them harder to inactivate during commercial processing. Previous studies of *Salmonella* thermal resistance focus on static conditions, overlooking the dynamic temperature and water activity changes that occur during actual food processing. The results of this current study show that properly designed dynamic experiments are a better way to accurately describe the *Salmonella* lethality during heat treatments, including pasteurization and drying. To develop effective mathematical models for pathogen inactivation in dynamic systems, it is crucial to incorporate dynamic temperature and moisture status conditions, as well as other relevant data, into the model. Simultaneous one‐step nonlinear regression offered several advantages over two‐step regression, including better representation of dynamic commercial processes, simpler experimental settings, and fewer experiments required. Future studies should focus on addressing the challenges in accurately monitoring water activity and moisture content changes of the food matrix during continuous thermal treatment.

## AUTHOR CONTRIBUTIONS

**Natoavina T. Faliarizao**: Conceptualization; formal analysis; writing—original draft; writing—review and editing; methodology. **Jemel Fanfan**: Investigation; methodology. **Narindra Randriamiarintsoa**: Formal analysis; investigation; writing—review and editing. **Muhammad Siddiq**: Supervision; writing—review and editing. **Teresa M. Bergholz**: Supervision; resources; writing—review and editing. **Kirk D. Dolan**: Writing—review and editing; supervision; resources.

## CONFLICT OF INTEREST STATEMENT

The authors declare no conflicts of interest.
